# Hearing Through the Patient’s Ears: Hearing Simulation for Counseling and Education

**DOI:** 10.3390/audiolres16030077

**Published:** 2026-05-22

**Authors:** Hailey A. Kingsbury, Sarah E. Kingsbury, Michael J. Cevette, Jan Stepanek, Edwar Habr, Hussein Itawi, Gaurav N. Pradhan

**Affiliations:** 1 Department of Otolaryngology-Head and Neck Surgery, Division of Audiology, Mayo Clinic, Scottsdale, AZ 85259, USApradhan.gaurav@mayo.edu (G.N.P.); 2 Aerospace Medicine and Vestibular Research Laboratory, Mayo Clinic, Scottsdale, AZ 85259, USA; stepanek.jan@mayo.edu; 3 Aerospace Medicine Program, Department of Internal Medicine, Mayo Clinic, Scottsdale, AZ 85259, USA; 4 Hi Dev Mobile Inc., San Mateo, CA 94403, USA; 5 Department of Biomedical Informatics, Mayo Clinic, Scottsdale, AZ 85259, USA

**Keywords:** hearing loss, simulation, amplification, communication partners

## Abstract

**Background/Objectives**: Hearing loss is frequently misunderstood by communication partners and healthcare providers of patients with hearing loss alike, leading to gaps in counseling, medical adherence, and empathy. Hearing loss simulation is a technique that can clarify patient experience by illustrating how hearing loss affects daily listening and speech discrimination. The current research explores the influences of a web-based hearing loss simulator on how hearing loss is understood by communication partners, audiologists, and medical providers/students. **Methods**: Using a convergent parallel mixed-methods design, 72 participants (communication partners, audiologists, and medical providers/residents) completed a guided trial with the hearing simulator, either one-on-one or in group settings, and subsequently completed a questionnaire regarding their experiences. The simulation trial included demonstrations of hearing loss and amplification across four listening environments: speech in quiet, in noise, at a distance, as well as listening to instrumental music. **Results**: Communication partners reported that they gained a better understanding of their partner’s hearing loss, with all expressing increased feelings of empathy towards their partner and individuals with hearing loss. When compared to audiologists, communication partners were significantly more surprised by the impact of hearing loss on speech understanding. Medical providers and students emphasized the value of the simulator as a counseling tool and highly recommended that other providers use the simulator with their patients. **Conclusions**: Simulating hearing loss can be beneficial for counseling purposes and medical training. These findings suggest that hearing loss simulation can enhance empathy and communication across clinical and educational contexts.

## 1. Introduction

Hearing loss is commonly misunderstood by both communication partners and healthcare providers, who often overestimate the degree of benefit provided by hearing aids. Even with amplification, individuals may continue to experience substantial difficulty in understanding speech, leading to communication breakdowns and frustration across home, social, and healthcare settings [1,2,3]. Audiologists likewise face challenges in effectively conveying the functional impact of hearing loss and the risks associated with untreated hearing loss without experiential demonstrations. Simulation is a method to address these issues by audibly demonstrating the impact of hearing loss on speech understanding across everyday environments. Techniques such as bandpass filters, video simulations, or virtual reality (VR) simulations have been shown to be effective for counseling patients and training medical students on the impact of hearing loss for decades [4,5,6]. However, there is limited research assessing the effects of a flexible, accessible simulator on the attitudes, empathy, and perspectives of individuals who frequently interact with patients with hearing loss.

### 1.1. Hearing Loss Prevalence and Amplification Uptake

Approximately 15% of American adults report some trouble hearing [7], and an estimated 37.9 million Americans (about one in nine) have bilateral hearing loss [8]. As one of the most common invisible disabilities, untreated hearing loss, especially comorbid with tinnitus, is associated with depression and anxiety [9]. Even after people are identified as hearing aid candidates, there is a long lag between hearing loss diagnosis and amplification adoption. On average, for patients who would benefit from hearing aids, the time between hearing loss diagnosis and hearing aid adoption is 8.9 years [10]. About 28.8 million U.S. adults could benefit from using bilateral hearing aids, but adoption rates are much lower at 39.1% [7,11]. Many patients report that they do not believe their loss is severe enough for amplification, despite receiving a professional recommendation to pursue hearing aids [12]. Self-perception and denial of hearing difficulty have been found to affect help-seeking behaviors, including hearing-aid uptake, use, and satisfaction with devices [13,14]. These attitudes, including motivation to pursue hearing aids, are often influenced by family members and friends [15,16]; individuals are more likely to seek help for their hearing loss if they experience social pressure from significant others to do so [17].

### 1.2. Effects of Hearing Loss on Communication Partners

Strain on interpersonal relationships is one of the most common and distressing effects of hearing loss [1,15]. In the context of this paper, the term “communication partners” will be used for individuals (e.g., spouses, family members, friends) interacting frequently with an individual with hearing loss. In a study evaluating the experiences normal-hearing spouses have due to their partners’ hearing loss, nearly all spouses reported exhaustion from repeating themselves (97%) and using raised voices (83%), and many expressed overall concern for their spouse’s safety (71%) [1]. Spontaneous remarks and interactional communication, integral to close, interpersonal relationships, are lost if one spouse cannot hear the other [3]. Additionally, communication partners often misunderstand the impact hearing loss has on speech understanding, confusing sound awareness (i.e., ability to detect the presence of sound) with the ability to discriminate speech clearly [3].

### 1.3. Perceptions of Hearing Loss by Medical Providers

Many medical providers are unaware of how hearing loss can affect patient compliance, follow-up care, and information retention, impacting overall health outcomes [18]. Patients with untreated hearing loss have lower adherence to medical direction, largely because of listening challenges and ineffective communication strategies in appointments, placing them at higher risk for adverse events and longer hospital stays [18,19]. Common healthcare practices such as scheduling appointments by phone, conducting visits in reverberant examination rooms, and using mouth coverings to reduce disease transmission can further impede communication for these patients. Moreover, limited provider training on the functional impact of hearing loss may contribute to health disparities [18]. These issues financially burden both the patient and the healthcare system, as they lead to higher expenditures and return to clinic due to misunderstandings of crucial medical directions [19]. Although audiologists receive specialized training in hearing healthcare, clinicians across all medical specialties should have a working knowledge of the effects of hearing loss to adapt their counseling strategies accordingly. Simulation-based training, especially following the COVID-19 pandemic, has become more widely utilized for medical training to improve practical skills and increase empathy for patients [20,21]. Improving provider awareness of the impacts of hearing loss may enhance patient outcomes through clearer in-clinic communication strategies and by encouraging patients to pursue amplification.

### 1.4. Perceptions of Simulators by the Disability Community

Any simulation of a disability is, at best, an approximation. Additionally, users will only ever be exposed to the simulated experience for a short time, meaning that no simulation can encapsulate the reality of a disability and lived experiences, including intersectional identities and environmental influence [22,23,24,25]. Another complaint disability activists have regarding simulation experiences is that they can invoke feelings of pity in participants, with some arguing that this increases stigmatization for the disability rather than addressing systematic, environmental, and behavioral barriers [26,27,28]. Additionally, some worry that since simulation represents one experience, it can lead to misunderstanding the diversity of disability [29]. Despite these limitations, some literature reveals that simulation can be effective and inclusive if the simulators rely upon counsel from the disability community and include their perspectives in design and delivery [25]. Simulation experiences are more impactful for healthcare providers if they are integrated throughout training and higher education rather than as a one-time experience [29].

The primary objective of this cross-sectional study is to assess whether exposure to a hearing loss simulator affects perceptions of hearing loss and understanding of its effects amongst those in frequent contact with individuals with hearing loss. It was hypothesized that communication partners would demonstrate greater increases in empathy and surprise compared with audiologists and medical providers, but all groups were expected to report perceived benefit from using the simulator.

## 2. Materials and Methods

### 2.1. Participants

The current protocol was approved by the Mayo Clinic Institutional Research Board (IRB) (ID: 25-004038). Participants (*n* = 72) included audiologists (*n* = 39), medical providers/students (*n* = 20), and communication partners of audiology patients (*n* = 13). Audiologists, audiology students, otolaryngologists, otolaryngology residents, nurse practitioners, and physician assistants were recruited to participate via email communication, grand rounds presentations, and conference series. Communication partners were recruited with information cards when accompanying patients to diagnostic hearing evaluation appointments.

### 2.2. Equipment and Calibration

**Figure 1:**
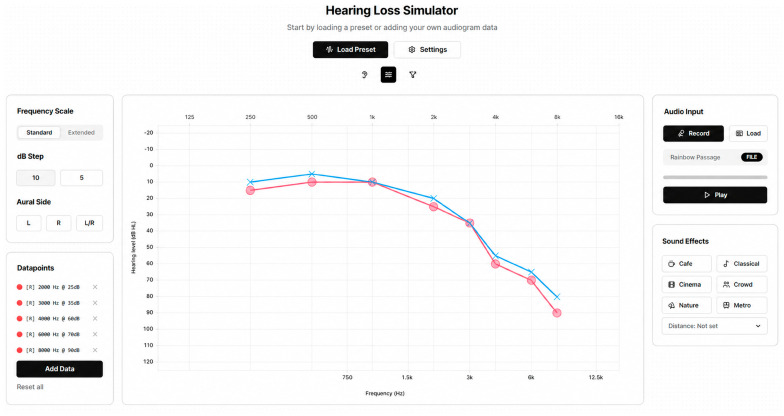
Hearing loss simulator interface. See Appendix A for feature details.

The hearing loss simulator (Hi Dev Mobile Inc., San Mateo, CA, USA) is a web-based platform that delivers a programmable, individualized simulations of hearing loss, with independent adjustment of each ear (Figure 1). Many hearing loss simulators have been developed without the perspectives of individuals with hearing loss [6]; therefore, it was important to the research team that development of the hearing loss simulator was guided by experiences of individuals with hearing loss. The current simulation software was conceptualized by an audiologist with hearing loss, and feedback for the next iteration of software development was obtained from individuals with hearing loss (*n* = 37). The simulator can demonstrate how gain from hearing aids would affect sound quality and intensity as heard through the hearing loss, allowing listeners to compare normal hearing, unaided hearing loss, and aided hearing profiles. The output for the hearing aid feature was approximated using a web-based hearing aid compression fitting algorithm based on the Desired Sensation Level (DSL) v5.0 prescriptive formula (Signal and Imaging Processing Lab, University of Texas at Dallas, Richardson, TX, USA). The simulator also allows background noise to be overlaid onto speech or audio input, with an adjustable signal-to-noise ratio. Additionally, the effects of distance on the intensity of speech can be modeled, based on the inverse square law. See Appendix A for further explanation of the hearing simulator menu features.

**Figure 2:**
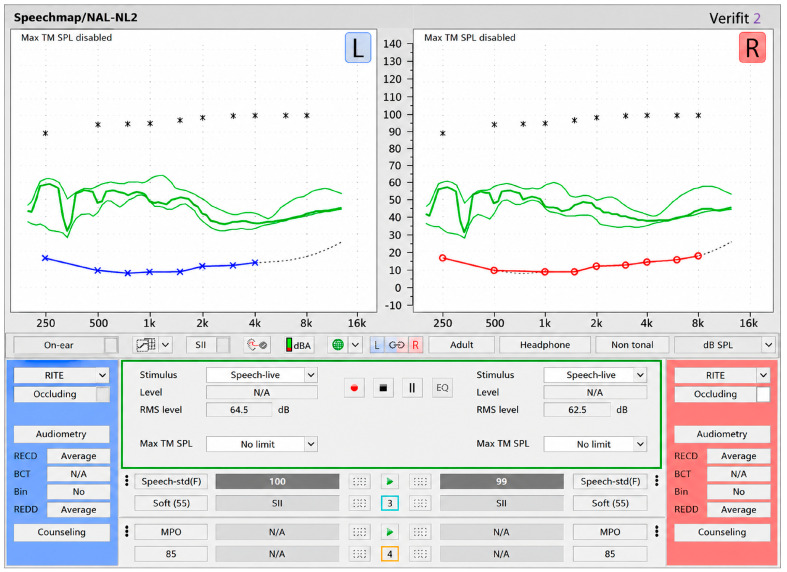
Verifit 2 Speechmap recorded on ear with live speech stimuli to verify output. Normal hearing thresholds were input, represented with red markings for the right ear and blue markings for the left ear. The green lines are the LTASS of the phonetically balanced speech sample that was played through the tablet via the headphones. The live RMS level shows that the speech sample was measured at an average conversational level (62–65 dB SPL). Asterisks (*) on the Speechmap represent uncomfortable loudness levels based on normal hearing audiogram.

In one-on-one demonstrations, the hearing simulator was deployed through a 14″ Windows 10 Pro tablet with circumaural headphones (SoundPlay, Tzumi Electronics, New York, NY, USA). Calibration of the simulator for average, conversation-level speech (65 dB HL) was completed with all thresholds of the hearing simulator set to 0 dB HL. Speech mapping measurements were obtained using the Audioscan Verifit2 (Audioscan, Dorchester, ON, Canada) with phonetically balanced recorded speech stimuli delivered via the hearing simulator software on the tablet and with the headphones used in the current study. Speech-live input was presented at conversational levels (approximately 62–65 dB SPL RMS), and the resulting output was measured at the ear reference point. Measurements were displayed using the NAL-NL2 prescription with on-ear speech mapping. The real-time speech spectra (long-term average speech spectra [LTASS] with dynamic speech range) were recorded for each ear, allowing verification of audibility across frequencies (Figure 2). It was confirmed that a volume setting of 20 (out of 100) on the tablet corresponded with an RMS level between 62 and 65 dB SPL consistently. During testing appointments, the tablet volume was set to the same level (20). Audibility was confirmed with biological equipment checks prior to research appointments.

During group presentations, the web-based simulator was demonstrated either in-person via a room speaker system or virtually through shared computer audio to illustrate its overall functionality and highlight differences between normal hearing, aided hearing, and unaided hearing loss profiles. Participants were informed, however, of the importance of calibrating the simulator with headphones for greater accuracy and validity in clinical appointment settings.

### 2.3. Procedures

All participants completed a trial with a web-based hearing loss simulator, either in one-on-one or group settings, and subsequently completed a questionnaire regarding their experiences. Most trials were completed in-person, but some trials for audiologists and students (*n* = 21) were conducted virtually. All trials of the software with communication partners were completed in-person at Mayo Clinic in Arizona with the Pro tablet and headphones.

The simulation included demonstrations of hearing loss and amplification across the following listening environments: speech in quiet, speech in noise, speech at a distance of 12 feet, and instrumental music. Audiologists and medical providers/students trialed the simulator using a sloping sensorineural hearing loss configuration (Appendix A). Communication partners trialed the simulator with their patient’s hearing loss configuration or the sloping sensorineural hearing loss preset configuration. Participants then completed a questionnaire incorporating both Likert-scale and free response questions regarding their experience with the simulator (some questions listed in Table 1).

### 2.4. Methods of Inquiry

The focus of this study was on understanding how exposure to a hearing loss simulator shapes attitudes, empathy, and perceptions of hearing loss among healthcare providers and communication partners, while also capturing perspectives on the lived experiences of individuals with hearing loss. A convergent parallel mixed-methods design was implemented [30], with quantitative and qualitative data being collected simultaneously through open- and close-ended questionnaires and interactive polling during presentations.

### 2.5. Data Analysis

#### 2.5.1. Quantitative Analysis

Seven constructs were represented in the Likert scale questions presented to communication partners, audiologists, and medical providers/students: empathy, understanding experiences of hearing loss, understanding challenges of hearing loss, realistic representation of hearing loss by the simulator, surprise by the impact of hearing loss, effectiveness of the simulator at representing hearing loss, and if they would recommend hearing healthcare providers use the simulator. Across all Likert-style questions, lower scores (1) were associated with maximal agreement (e.g., “Yes, a great deal”; “Extremely realistic”) and higher scores (5) were related to minimal agreement, or disagreement (e.g., “No, not at all”; “very ineffective”). After every testing session, scores derived from the questionnaires were recorded in Qualtrics 2026 version (Qualtrics LLC, Seattle, WA, USA) and then exported to Excel spreadsheets (Version 2510, Microsoft Corporation, Redmond, WA, USA). Statistical analyses were performed using R Statistical Software (v4.5.0; R Core Team 2025, Vienna, Austria).

#### 2.5.2. Qualitative Analysis

Qualitative data was collected from open-ended answers on the written or web-administered questionnaire in response to the question “How did using the hearing loss simulator affect your perspective on hearing loss and its impact on daily life?” Representative comments that participants made during their research appointments were transcribed by the primary data abstractors. A semantic, inductive approach was used to analyze text responses from the questionnaires using thematic analysis [31,32]. In their seminal article, Braun and Clarke created an approach to thematic analysis consisting of six steps: familiarization with data, generating initial codes, searching for themes, reviewing these themes, defining and naming themes, and producing a report [31]. Each abstractor manually coded the qualitative responses independently. Codes were first formulated based on the content of the responses across each of the open-ended questions. They were then refined and categorized to create more valuable and comprehensive categories. Codes were grouped iteratively into themes, completing the process of thematic analysis [33]. Results were then merged for interpretation and comparison. Discrepancies were resolved after initial coding through a series of meetings between abstractors.

## 3. Results

### 3.1. Quantitative

**Figure 3:**
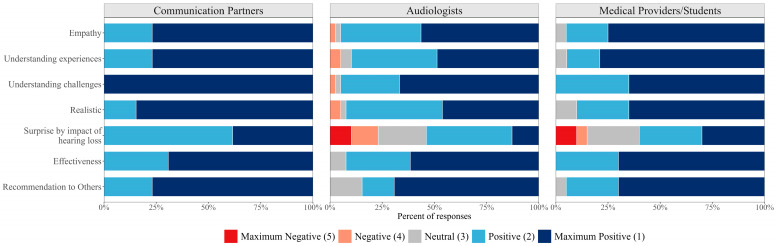
Stacked bar chart of Likert scores of shared constructs between communication partners, audiologists, and medical providers/students.

Communication partners, audiologists, and medical providers/students all rated the simulator as an effective tool to encourage understanding of hearing loss and recommended its use to others (see Table 1 and Figure 3). Regarding its utility as a counseling tool, providers reported the simulator would have good utility in getting individuals interested in amplification. Across communication partners, 92% expressed they had more realistic expectations for hearing aids following trialing the hearing simulator. There was unanimous agreement amongst communication partners that the hearing loss simulator helped them better understand the challenges experienced by individuals with hearing loss (M = 1, *SD* = 0).

#### Group Comparisons

**Figure 4:**
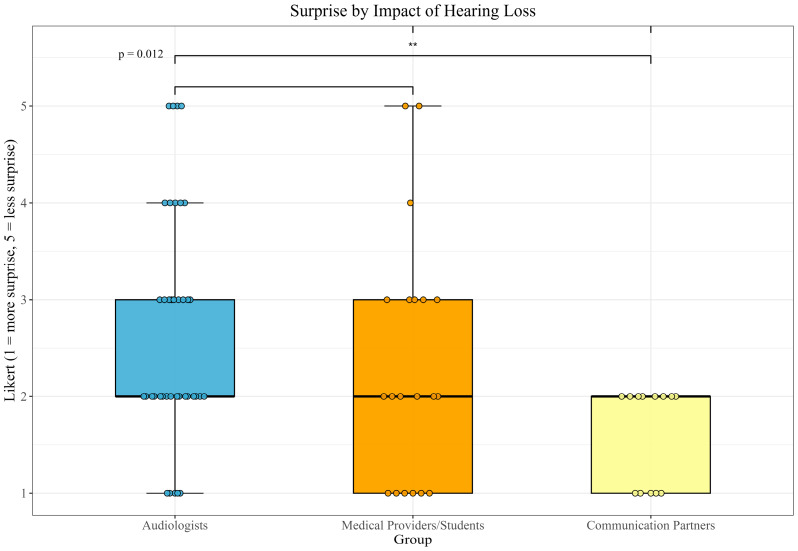
Boxplot of significant difference between surprise by impact scores between audiologists and communication partners (1 = more surprise, 5 = less surprise). Significance level (*p* < 0.05) indicated by double asterisk (**).

Shapiro–Wilk tests for normality were performed for each feature by group (Audiologists, Medical Providers/Students, and Communication Partners). Non-parametric comparison testing was conducted using Kruskal–Wallis tests followed by post hoc pairwise Mann–Whitney U tests (implemented as Wilcoxon rank-sum tests in R) with Bonferroni correction. Kruskal–Wallis testing showed a significant difference in feelings of surprise at the impact of hearing loss on speech understanding across groups (χ^2^ = 7.51, df = 2, *p* = 0.023) (Figure 4). The effect size across the three groups, quantifying the magnitude of difference between groups, was moderate ($\eta_{H}^{2}$ = 0.085). Post hoc pairwise Mann–Whitney U tests indicated that Audiologists (*n* = 39) and Communication Partners (*n* = 13) differed significantly (*U* = 117.50, adjusted *p* = 0.007) in their level of surprise at the impact of hearing loss (*r* = 0.43), indicating a moderate effect, with Communication Partners reporting greater surprise. For all other constructs, mean scores were less than 2, indicating maximal to moderate positive scores across all three groups. Other pairwise comparisons were not significant.

### 3.2. Qualitative

Thematic analysis was conducted on the short responses to open-ended questions from the communication partners, audiologists, and other medical providers/students.

#### 3.2.1. Communication Partners

There were four themes identified in the written and oral statements made by communication partners related to how the simulator shaped their perspectives (Table 2): (1) improved understanding of the functional impact of hearing loss, (2) increased empathy, (3) shifts in personal perspective, and (4) more realistic expectations regarding the utility of hearing aids for hearing loss. Communication partners frequently noted that the simulator reminded them of the need to adapt their communication behaviors to their partner’s hearing needs, as one participant acknowledged, “It is not how I communicate with most other people in my life.” Many reported the simulator illuminated previously unrecognized aspects of their loved one’s experience with hearing loss (e.g., “Much more aware of the impact”), while others expressed newfound or deepened empathy (e.g., “Made me understand my husband’s disability better”). Several partners articulated intentions to modify their communication practices, noting the simulation enhanced their motivation to support individuals with hearing loss more effectively. Partners also recognized that hearing aids improve audibility but do not restore normal hearing, especially in situations with loud background noise, leading to more realistic expectations following the simulator experience. In a couple of cases, the communication partner wondered if they could benefit from amplification (e.g., “I might need hearing aids”), indicating that the difference between the speech and sounds filtered through the hearing loss and aided hearing filters was meaningful to them.

#### 3.2.2. Audiologists

There were four themes identified in the written and oral statements made by audiologists related to how the simulator shaped their perspectives (Table 3): (1) Perceived value as a counseling tool, (2) increased empathy for patients, (3) situational understanding across environments, and (4) validation of existing clinical knowledge. The most prominent patterns were how audiologists saw value in simulation as a counseling tool and had increased empathy for the patient’s experience. Audiologists noted that different environmental listening situations (e.g., background noise, music, nature sounds) magnified the difficulties patients can have with speech understanding. Several providers noted that the simulator could help recalibrate expectations for amplification by demonstrating that hearing aids improve audibility, but do not restore normal hearing. This was especially noted when listeners tried to parse speech from background noise or appreciate music with the hearing aid filter (e.g., “The background noise simulation made it very clear why patients struggle in restaurants”). Finally, some audiologists wrote or noted verbally that the simulation did not necessarily teach them anything new about hearing loss, but rather, reinforced what they frequently counsel patients about, validating their existing knowledge (e.g., “It did not change my perspective but I am also an audiologist and this information is not new to me”).

#### 3.2.3. Other Medical Providers/Students

There were three themes identified in the written and oral statements made by medical providers/students related to how the simulator shaped their perspectives (Table 4): (1) the value of simulation for counseling and education, (2) simulation as a tool to build empathy and understanding, and (3) the importance of experiential learning. Medical providers/students emphasized increased understanding of the lived experience of their patients with hearing loss. Even though nurse practitioners, physician assistants, otolaryngology residents, and students are not practicing audiologists, they still noted that the simulator would be an effective tool to encourage better communication practices for their patients (e.g., “I think it’s a great way to show people how hearing loss affects everything in someone’s daily life”). They also noted that experiential learning, or tangible teaching where concepts are shown instead of told, can be far more memorable for patients, their communication partners, and students (e.g., “Brought more specific aspects into focus that aren’t as easy to replicate on our own”).

## 4. Discussion

This study aimed to offer insight into the expectations, knowledge, and experiences of communication partners, audiologists, and other health providers regarding hearing loss. Both quantitative and qualitative findings may be broadly understood within social perspective-taking and experiential learning frameworks [34]. Across the three groups of participants, there were some overlapping themes that emerged during thematic analysis of the qualitative responses and were supported by the results of the quantitative analysis. These common themes were understanding hearing loss experiences, the impact of simulation on empathy, and the utility of simulation for counseling and education, particularly regarding setting expectations for amplification.

### 4.1. Understanding Hearing Loss Experience

Participants had increased awareness of how hearing loss affects communication in real-world environments following demonstrations with the simulator. Communication partners exhibited the greatest degree of change in perceptions towards hearing loss, as supported by quantitative findings showing significantly higher surprise ratings relative to providers. Upon reflection of their experience with the simulator, communication partners reported greater awareness of what was not audible to their partner with hearing loss (e.g., “I have a better understanding of what he is not hearing”). Participants also noted that the simulation highlighted how certain environments, particularly those with background noise, may heighten listening challenges (e.g., “How the ambient noise impacts the ability to clearly focus on heaving a conversation. [It was] much more difficult with a hearing impairment”). This finding aligns with the prior literature reporting that communication partners often underestimate the impact of hearing loss and may believe they are accommodating communication barriers in conversation with their loved ones with hearing loss [2]. When ineffective communication strategies remain unaddressed, repeated communication breakdowns can become isolating and burdensome to spouses and family members of individuals with hearing loss, leading to frustration and resentment over time [1,3]. Increased understanding of the difficulties hearing loss can present for speech understanding, especially in the presence of background noise, could influence communication partners to adopt clearer, face-to-face communication techniques that help hearing aid users and non-users alike.

When working with patients and their families, counseling appropriate expectations is a central, but often challenging, component of care. While audiologists expressed the least amount of surprise regarding the impact of hearing loss on speech understanding, they reported that the simulator increased their understanding of the experience and listening difficulties of individuals with hearing loss. Audiologists more often reported that the simulator reinforced their existing knowledge of hearing loss (e.g., “Very powerful tool to quickly demonstrate what we know to be true about hearing loss and its impact”). This suggests a gap between audiologists’ understanding of hearing loss and the extent to which patients and communication partners grasp knowledge during clinical appointments. This finding builds on prior studies that say understanding a patient’s perspective and lived experience are more effective for medical adherence than information-based counseling alone [35]. Understanding the communication challenges faced by patients with hearing loss can help motivate providers to use more effective communication strategies [36], such as facing the patient when speaking, using visual cues, and the teach-back method.

### 4.2. Impact of Simulation on Empathy

Along with helping with skills-based practice, simulators can aid in developing intangible qualities that impact patient care, like empathy. All groups noted deepened emotional understanding of individuals with hearing loss. While there were no significant differences between the three groups in their scores reporting effects on empathy (“Do you think this simulator affects the empathy with how you respond to people with hearing loss?”), mean empathy ratings were clustered towards maximal agreement. This indicates that even amongst experienced hearing healthcare providers, immersive exposure to hearing loss deepened their ability to understand and share the feelings of their patients. These findings extend prior work done with VR simulations in healthcare training environments, demonstrating that web-based simulation platforms can produce empathetic effects [5]. The most effective simulations for developing empathy in medical students are those that require the learner to “stand in the patient’s shoes” [37]. Increases in empathy serve practical purposes; McLaughlin et al. [5] found that when providers experienced hearing loss through simulation, it motivated them to make more effective communication environments in the clinic.

For communication partners, building empathy can have a real impact on the trajectory of their loved ones’ hearing healthcare journey. A person’s hearing loss also has a psychosocial effect on the person they primarily communicate with, and empathetic alignment is critical for coping and effective rehabilitation [15]. Since communication partners tend to recognize effects of hearing loss before the patients themselves do, increased feelings of empathy in communication partners can improve the uptake of hearing rehabilitation and reduce resistance to intervention [38]. Prior to leaving a research appointment, one communication partner commented, “It was really enlightening to see where the other person is at. I love being able to understand things from this standpoint… The question about empathy is so spot-on… because you have built-in assumptions, and it helps with seeing it in a different way.” Hearing loss is relational, and communication partners can make assumptions about sound perception if they do not understand how hearing loss, competing noise, and distance all can affect understanding, leading to frustration and strain on the relationship [15]. Individuals are more likely to adopt hearing aids if they perceive that their significant other is supportive of seeking hearing rehabilitation [39], with greater social support and aligned coping resulting in deepened empathy.

### 4.3. Utility of Simulation for Counseling and Education

Education about the causes and effects of hearing loss can help communication partners better support their loved ones, and medical providers better serve their patients. All scores on the Likert questions assessing if communication partners would recommend the simulator be used in audiology appointments indicated moderate to maximal agreement. Even though family member support is associated with higher prevalence of seeking hearing rehabilitation [39], third party roles are often overlooked in audiology appointments. In a qualitative study that reviewed videos from hearing aid consultation appointments, family members often indicated interest in sharing their experiences and participating in appointments (e.g., replying to questions the audiologist would address to the patient, serving as an intermediary in conversation, expanding upon patient answers), but they were rarely given time to contribute [40]. The knowledge gap between audiologists and communication partners is evidenced in the significant difference between median scores regarding surprise at the impact of hearing loss. The conversational effects of hearing loss can significantly affect primary communication partners, though, and involving them in hearing loss treatment decisions is mutually beneficial [41]. Having a tool that simulates hearing loss for a listener with normal thresholds indicates that their understanding of the lived experience of hearing loss is crucial for the success of their partner. This is aligned with the American Speech-Language-Hearing Association’s (ASHA) Preferred Practice Patterns recommending family-centered care [42]. Audible simulations could enable this quality of care.

Both audiologists and other medical providers emphasized the value of a hearing simulator for educating patients, communication partners, and students alike. Simulation has long been used in healthcare to optimize safety, quality, and efficiency of care in low-stakes training environments [43]. Even so, students in pre-professional healthcare programs receive limited training on how hearing loss affects quality of life in affected patients [44]. In Communication Sciences and Disorders healthcare training programs, simulators can teach speech-language pathology and audiology students about the importance of aural rehabilitation [45]. In these cases, students must be reminded that a hearing loss, in most cases, is a progressive disability that a person adapts to throughout its course. Just as the hearing loss had to be adapted to, hearing aids have an adjustment period, requiring the user and communication partners to develop new communication strategies. The current study extends the work that has shown hearing loss simulators are effective tools for counseling and training students about the impact of hearing loss on speech discrimination, especially in medical environments [46]. Medical providers and students in this study ranked the simulator as highly effective and reported it helped them better understand the experience of individuals with hearing loss. This is especially important for medical students in specialties outside of audiology, since there is a high prevalence of hearing loss in the general population, but over half of all pre-professional healthcare students reported that they do not feel confident enough in their knowledge of hearing loss to make a referral to audiology [44]. Increased understanding of the impacts of hearing loss could help medical students identify hearing loss as a condition that affects overall quality of life.

#### Expectations for Amplification

Another important aspect providers mentioned while counseling is setting realistic expectations for amplification. Across responses, there was emphasis on the importance of framing hearing aids as a “tool” or “support” for those with hearing loss rather than as a “fix” or “solution” to restore hearing. These findings reinforce the relational complexity of hearing-loss counseling: providers are tasked with simultaneously managing patient readiness, communication partner misconceptions, and sociocultural beliefs about hearing aids. One provider commented that the simulation tool would be “particularly valuable for counseling family members,” who often hold assumptions about what hearing aids “should” accomplish. Communication partners expressed that simulation exemplified the benefits and limitations of amplification, with 92% reporting that they had more realistic expectations for amplification following trialing the hearing simulator. For instance, the directional microphones in hearing aids are most effective in face-to-face conversational settings of 3–6 feet; many communication partners assume that purchasing hearing aids will make up for all communication deficits, including speaking across significant distance and over background noise. When they were exposed to the decrement in intensity a 12-foot distance could present, they noted that the hearing aid made things only slightly more audible (e.g., “The hearing aids made things louder, but it was still quite quiet”). Using the simulator with the distance filter could justify why hearing aid accessories, like a remote microphone, could significantly improve a patient’s speech recognition across distance and over background noise [47]. Many communication partners were impressed by the degree of benefit the hearing aid provided for understanding speech, but there were comments on sound quality differences, especially with music (e.g., “I know he is missing out on all those higher frequencies now. He was a bass and euphonium player, and I now see what he is missing”). Many communication partners are highly supportive of amplification, as previous research has found that post-fitting, both the hearing aid user and the communication partner report improvement in quality of life [48]. However, unrealistic expectations for amplification can place further stress on patient–partner communication.

Often, communication partners focus on the role of technology in hearing rehabilitation (i.e., purchasing hearing aids), but may have unrealistic expectations for devices. Without appropriate education from an audiologist, they may not realize that making changes to their communication practices is essential to optimize this technology. While hearing aids make sound significantly more accessible to users, they operate best when the conversation partner is within 3–6 feet of the user and facing them. There is shared responsibility that comes with repairing nonfunctional communication behaviors (e.g., speaking from different rooms, talking over the television, not catching attention prior to speaking) that requires both the patient and communication partner to amend their behaviors [49]. This was reflected in some of the communication partner comments after listening to speech over background noise and instrumental music through the hearing aid filter (e.g., “I understand better what my wife is experiencing and how hearing aids will sound to her”). Addressing this gap in understanding between audiologists and the people they serve is essential to not only improve transfer of information but also improve the quality of life and satisfaction with devices experienced by audiology patients.

### 4.4. Validity and Limitations

Methods to enhance validity and develop a comprehensive understanding of the impacts of hearing loss simulation were implemented throughout the study design, data collection, and data interpretation. Triangulation, or the use of multiple methods and data sources to corroborate findings, was achieved by drawing from both qualitative and quantitative data sources to corroborate findings and enhance internal validity [50]. Purposeful sampling was used across healthcare providers, patients, and communication partners to provide varied participant perspectives from differing backgrounds and roles. This supported a more comprehensive understanding of experiences with hearing loss and simulation-based education. To promote transferability, the study includes detailed descriptions of the research materials and procedures so the same process could be replicated in graduate programs, private practices, or other hearing healthcare settings. Coding triangulation was also employed in this study, as two researchers separately coded qualitative responses before coming together to compare their interpretations. This added layer of comparative analysis strengthens the dependability and confirmability of findings.

Reflexivity, or researcher reflection upon their relationship to the phenomenon they are studying, was maintained throughout the study, but the researchers’ backgrounds and values will naturally affect interpretation of the qualitative results. As the primary researchers for this study were both licensed audiologists with normal hearing, they have received extensive education regarding the causes and effects of hearing loss, but do not represent the target audience of this tool. Additionally, the novelty of the simulator for some communication partners could lead to a bias towards reporting more positive attributes of the tool instead of critically examining the weaknesses of the simulator.

There were inherent limitations to this study, chief amongst them the issue of using a simulator to approximate a real experience. Participants were reminded that the simulator, including the hearing loss filtering and hearing aid features, is a model. Thus, it is impossible to fully replicate all experiential elements of hearing loss; however, there are elements of hearing loss that providers would expect to be represented in a medical simulator. Participants were encouraged to critically reflect on the representation of hearing loss. Amongst audiologists, a common report was that while the simulator showed decrements in intensity across frequency bands, it did not replicate the distortion and reduced clarity of speech that audiologists know is an effect of sensorineural hearing loss [51]. Additionally, since this study consisted of a single appointment, the permanence of what was learned from the simulation could not be assessed. While medical providers report intentions to apply their learning in clinical work [45], less is known about long-term behavioral changes following simulation experiences. Longitudinal research is needed to evaluate the impact of simulation-based training and counseling on individuals’ beliefs and behaviors.

Future directions include adding a word recognition score (WRS) slider to improve the authenticity of the simulator. Since hearing loss affects both volume and clarity, adding this layer to the simulator will aim to better represent the auditory experiences of people with hearing loss. It is important that the perspectives of those with hearing loss are incorporated in the next software version. To achieve this goal, we have presented the simulator to patients from the Mayo Clinic Division of Audiology (*n* = 37) who had diagnostic hearing evaluation appointments. These patients ranged in age and degree of hearing loss, and their feedback is being incorporated into the next iteration of the software. Individuals with hearing loss have embodied knowledge of hearing loss, which many audiologists and other hearing healthcare professionals do not; thus, any teaching tool needs to be constructed with input from those with the actual experience of the disability being simulated [22,25]. While there are challenges that emanate from playing auditory approximations of hearing loss and hearing aid amplification to someone with an existing hearing loss, we know it is essential to solicit and use the feedback from the population whose experience we are trying to elucidate.

## 5. Conclusions

Experiential feedback from communication partners, audiologists, and other medical providers, including students/residents, emphasize that hearing loss simulation is a promising tool to enhance empathy, recalibrate expectations, and support education for both healthcare providers and communication partners. For communication partners, simulation has utility in setting realistic expectations for hearing aids and could encourage earlier adoption of amplification. In clinical settings where a hearing aid fitting demonstration is not possible, simulation may also provide patients and families with practical approximations of aided hearing. For medical providers and students, simulation offers a helpful way to transfer knowledge from audiologists to learners, with minimal training time and maximal impact. Audiologists should feel encouraged to use hearing loss simulation in more of their routine diagnostic and hearing aid evaluation appointments. With these demonstrations, communication strategies should be taught, including clear speech techniques, facing patients and their partners, ensuring good lighting in examination rooms, and incorporating meaningful pauses around words requiring emphasis. In this way, the simulator can facilitate aural rehabilitation and encourage effective communication. 

## Figures and Tables

**Table 1:** Means and standard deviations from Likert-scale question scores (1 = maximum agreement, 5 = minimal agreement).

Question	Mean Likert Scores (M) and Standard Deviations (*SDs*)		
	Communication Partners	Audiologists	Other Medical Providers/Students
Do you think this simulator affects the empathy with how you respond to people with hearing loss?	M = 1.23; *SD* = 0.43	M = 1.51; *SD* = 0.68	M = 1.30; *SD* = 0.57
Did using the Hearing Simulator help you better understand the experiences of your patients/communication partner with hearing loss?	M = 1.23; *SD* = 0.44	M = 1.67; *SD* = 0.81	M = 1.26; *SD* = 0.56
Did the simulator help you understand the challenges faced by individuals with hearing loss?	M = 1; *SD* = 0	M = 1.41; *SD* = 0.68	M = 1.35; *SD* = 0.49
With your understanding of hearing loss, how realistic did the hearing loss simulator feel?	M = 1.15; *SD* = 0.38	M = 1.67; *SD* = 0.77	M = 1.45; *SD* = 0.69
To what extent were you surprised by the impact of hearing loss on listening?	M = 1.62; *SD* = 0.51	M = 2.67; *SD* = 1.18	M = 2.35; *SD* = 1.27
How would you rate the overall effectiveness of the simulator at simulating hearing loss?	M = 1.31; *SD* = 0.48	M = 1.46; *SD* = 0.64	M = 1.30; *SD* = 0.47
How likely are you to recommend using the simulator to other audiologists/healthcare providers/communication partners?	M = 1.23; *SD* = 0.44	M = 1.46; *SD* = 0.76	M = 1.35; *SD* = 0.59

**Table 2:** A thematic summary of communication partner responses to “How did using the simulator affect your perspective on hearing loss and its impact on daily life?”.

Theme	Description	Codes	Example Participant Quotation	Frequency ^1^
1. Understanding Impact of Hearing Loss	Partners expressed increased awareness of how hearing loss affects communication, attention, and engagement across real-world environments.	Hearing loss impact; Hearing loss daily life; Understanding hearing loss; Disability	“How the ambient noise impacts the ability to clearly focus on having a conversation—much more difficult with a hearing impairment.”	*n* = 5
2. Increased Empathy	Partners reported deeper empathy and recognition of the emotional challenges associated with hearing loss following the simulation	Empathy; Understanding	“It really opened my perception. I will be very good in the future to understand what my wife is experiencing.”	*n* = 5
3. Perspective Change	Partners reported a shift in their conceptual understanding of hearing loss, altering prior assumptions about what their loved ones can or cannot hear.	Perspective change; Patient perspective; Realistic	“It was great to get a realistic idea of what family members can hear.”	*n* = 4
4. Utility of Hearing Aids with Realistic Expectations	Partners gained clearer expectations about amplification, recognizing both its benefits and limitations and adjusting unrealistic expectations about hearing aids “fixing” hearing loss.	Sound difference; Understanding hearing aids	“I understand better what my wife is experiencing and how hearing aids will sound to her.”	*n* = 3

^1^ Responses could reflect multiple thematic elements, so a single response could be assigned to more than one theme.

**Table 3:** A thematic summary of audiologist responses to “How did using the simulator affect your perspective on hearing loss and its impact on daily life?”.

Theme	Description	Codes	Example Participant Quotation	Frequency ^1^
1. Perceived value as a counseling tool	Audiologists viewed the simulator as an effective tool to convey communication challenges to patients and families.	Counseling tool; Helpful	“Hearing the simulations for background noise and distance was powerful. These could be so helpful in counseling family members.”	*n* = 8
2. Increased empathy for patients	Audiologists developed deeper emotional and cognitive appreciation of their patients’ lived experiences.	Empathy; Patient perspective; Everyday life	“It definitely improved empathy for what those patients are going through.”	*n* = 7
3. Situational understanding across environments	Experiencing loss in noise, distance, and music highlighted how context magnifies impairment.	Background noise; Distance; Environment; Soundscape differences; Music appreciation; Nature	“The background noise simulation made it very clear why patients struggle in restaurants.”	*n* = 7
4. Validation of existing clinical knowledge	Providers noted that the simulation reinforced knowledge they already held about hearing loss challenges.	Confirmation; Alignment with prior knowledge	“Very powerful tool to quickly demonstrate what we know to be true about hearing loss and its impact.”	*n* = 2

^1^ Responses could reflect multiple thematic elements, so a single response could be assigned to more than one theme.

**Table 4:** A thematic summary of other medical provider/student responses to “How did using the simulator affect your perspective on hearing loss and its impact on daily life?”.

Theme	Description	Codes	Example Participant Quotation	Frequency ^1^
1. Value of simulation for counseling and education	Providers/students viewed the simulator as an effective tool to convey communication challenges to patients and families.	Counseling tool; creating understanding; Helpful	“Very effective demo. Will be great to explain to family members of patients with hearing loss.”	*n* = 5
2. Simulation as a tool to build empathy and understanding	Providers/students indicated increased empathy for their patients experiences and felt that this tool could increase empathy in others	Empathy; Patient perspective; Awareness of burden	“Increased empathy.” “Great insight to how to create more understanding in a communication partner.”	*n* = 4
3. Importance of experiential learning	Providers/students reported that explaining complex experiences can be challenging without immersive examples	Replication of hearing loss experience; Effective demonstration; Explanation	“Brought more specific aspects into focus that aren’t as easy to replicate on our own.”	*n* = 4

^1^ Responses could reflect multiple thematic elements, so a single response could be assigned to more than one theme.

**Figure A1:**
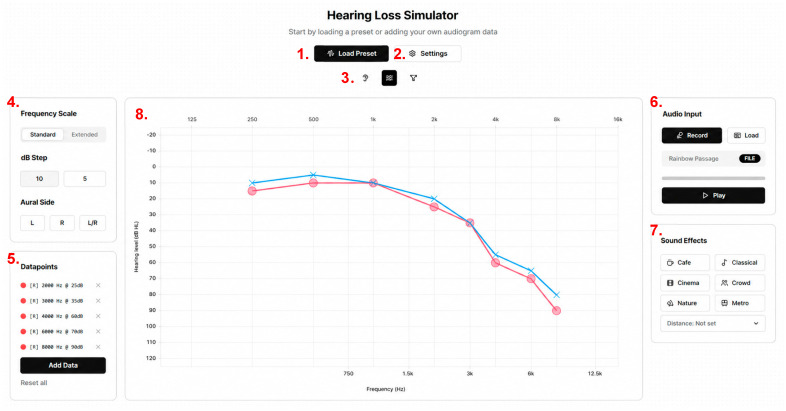
Hearing Simulator Parameters. 1. Load Preset: This is a curated set of common audiometric configurations. Choose one to load sample thresholds. 2. Settings: Change the theme between light or dark mode. 3. Audio Filter Menu: Adjust the audio filtering or audiometric display. The ear applies prescriptive gain to the hearing loss. The lines adjust the audiogram display, by displaying or removing the lines connecting datapoints. The funnel removes all hearing loss and prescriptive gain filters from the audio file, resetting to “normal.” 4. Audiogram Menu: Change the intensity in dB HL (*y*-axis) and frequency in Hz (*x*-axis) scaling. Change the aural side for plotting and display of audiometric thresholds. 5. Datapoints Menu: Displays entries for plotted data. Add or delete typed data to be graphed. Reset to set all thresholds to 0 dB HL. 6. Audio Input Menu: Change the audio input from a selection of preset options or by uploading or recording audio files. 7. Sound Effects Menu: Add different types of background noise, with adjustable volume, behind the audio signal. Adjust the distance from the audio source. 8. Audiogram: Display of plotted audiometric thresholds, with red indicating the right ear and blue indicating the left ear.

## Data Availability

The raw data supporting the conclusions of this article will be made available by the authors on request.

## Abbreviations

The following abbreviations are used in this manuscript:

VR	Virtual Reality
dB HL	Decibels Hearing Level
dB SPL	Decibels Sound Pressure Level
DSL	Desired Sensation Level
LTASS	Long-Term Average Speech Spectra
NAL-NL2	National Acoustic Laboratories’ second generation gain prescription
RMS	Root Mean Square

## Appendix A

The hearing simulator (Hi Dev Mobile Inc., San Mateo, CA, USA) has several adjustable parameters, including audiometric thresholds, audio input, and background noise.
